# Letter to the Editor: Predicting intraoperative blood loss risk in severe lumbar disc herniation patients undergoing PLIF: a multicenter cohort study using ensemble learning

**DOI:** 10.1097/JS9.0000000000003457

**Published:** 2025-10-08

**Authors:** Shiquan Pan, Long Jun Chen

**Affiliations:** a Department of Orthopedics, Yulin Hospital of Integrated Traditional Chinese and Western Medicine Orthopedics, Yulin, China; b Department of Spine Surgery Center, Ningbo Yinzhou Zhedong Orthopaedic Hospital, Ningbo, China

We read with interest the recently published article on the development of the IBLED-LDH model, an ensemble machine learning framework designed to predict intraoperative blood loss (IBL) in patients with severe lumbar disc herniation (LDH) undergoing posterior lumbar interbody fusion (PLIF)^[1]^. While the authors should be commended for leveraging large-scale, multicenter data and modern ML techniques to address personalized surgical risk stratification, several methodological and conceptual concerns merit further discussion.

The definition of “excessive IBL” in this study is based on a composite outcome: (1) IBL exceeding the 75th percentile (>400 mL), (2) a hemoglobin drop of ≥10 g/dL, or (3) intraoperative blood transfusion. However, these thresholds appear arbitrary and highly context-dependent. The 400 mL cutoff lacks external validation across different institutions, where surgical and anesthetic practices may vary substantially. Hemoglobin changes can be influenced by preoperative hydration status or hemodilution, while transfusion decisions are often shaped by provider preference or institutional policies. These factors may introduce considerable heterogeneity and potential label bias into model training.

Although the feature selection methods applied (e.g., LASSO and Boruta) are methodologically sound, the inclusion of variables such as “preoperative hospitalization days” and “duration of clinical course” raises concern. These factors may reflect institutional workflows or disparities in healthcare access rather than intrinsic patient physiology. Their inclusion risks conflating procedural inefficiency with biological bleeding risk. Similarly, while the statistical association between preoperative albumin and IBL may be significant, it likely reflects general frailty or nutritional status rather than a direct mechanistic link to bleeding.

Despite the use of 10-fold cross-validation and Bayesian optimization, the evaluation of 70 model combinations across overlapping feature sets raises the likelihood of overfitting. Furthermore, although four geographically stratified validation cohorts were employed, all data originated from the same registry (DSDC2024) and time frame (2015–2022). Without independent temporal or institutional validation for instance, from a different hospital network or a prospective cohort the model’s generalizability in real-world settings remains uncertain.

The authors utilized decision curve analysis to show that their ensemble model outperforms “treat-all” or “treat-none” strategies. However, they did not benchmark performance against comparable risk assessment tools^[2,3]^ or surgeons’ clinical estimates of bleeding risk. Without such direct comparisons, the incremental value of this complex model over standard clinical judgment is unclear.

Finally, we would like to express our sincere appreciation to the authors for their efforts in addressing the important issue of IBL in severe LDH patients undergoing PLIF. This study provides a valuable foundation. Nevertheless, we raise these questions in the spirit of constructive dialogue, and we encourage future work to explore prospective validation, refinement of model inputs, and stronger alignment with clinical decision-making pathways. The article was written in accordance with the TATIN guidelines^[4]^.

## Data Availability

Not applicable.
